# Supplementary material for: Cholera toxin remodels intestinal immunity and suppresses systemic metabolism
Source: bioRxiv. 2026 Oct 6:2026.09.29.755509. Preprint. [Version 1] doi: 10.64898/2026.09.29.755509 (PMC13691948; doi:10.64898/2026.09.29.755509)
Supplement: Supplementary file 6 [file media-6.pdf]

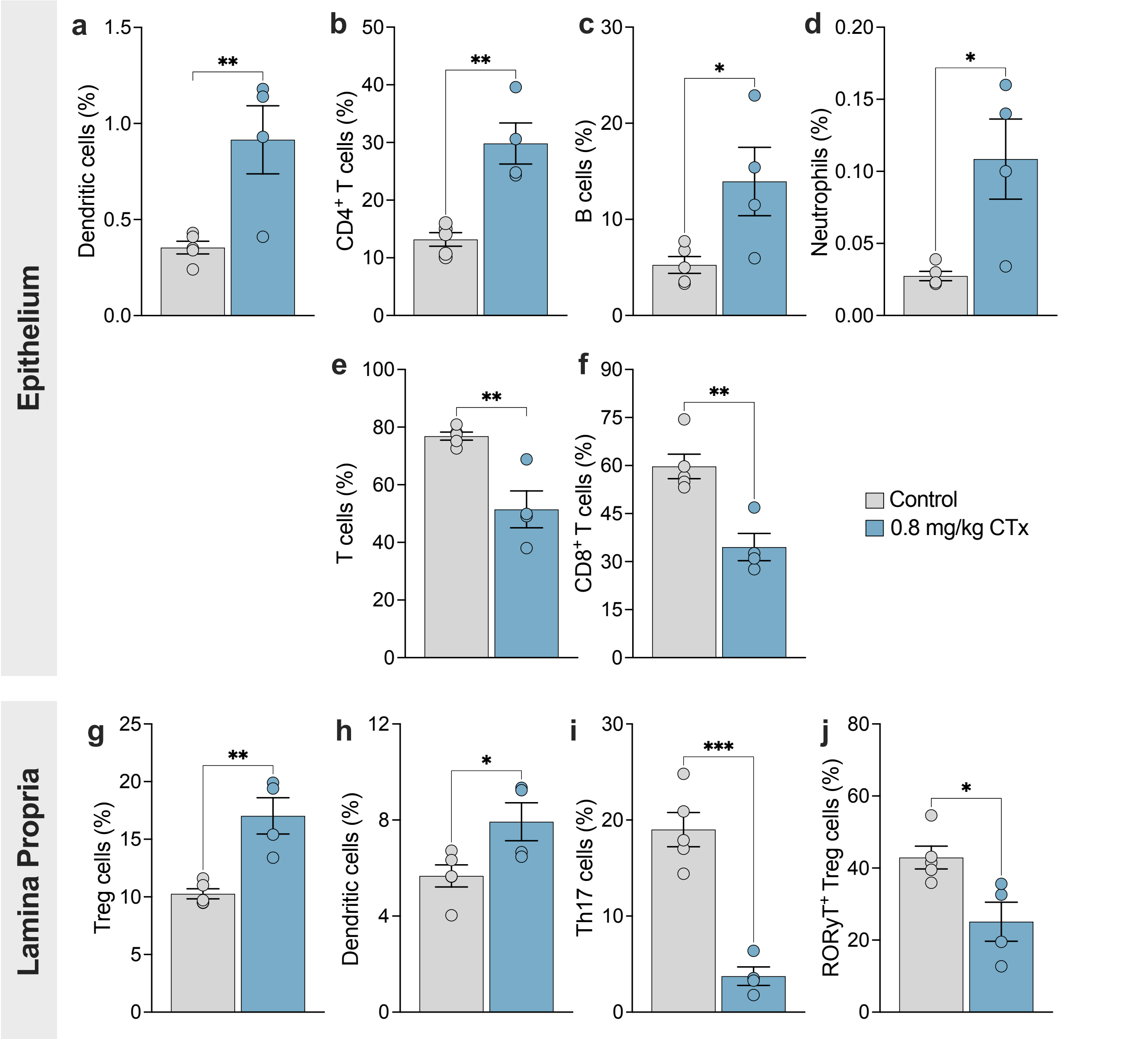

Supplementary Figure 5 | CTx remodels immune cell composition in the small intestine of C57BL/6 female mice. Small intestines from C57BL/6 female were collected 24 h after a single oral exposure to cholera toxin (CTx), enzymatically dissociated, and analyzed by full-spectrum flow cytometry (n= 4-5 mice per group). In the intestinal epithelium, CTx changed the frequency of **(a)** dendritic cells ($CD11c^+$ MHC-II^+), **(b)** $CD4^+$ T cells ($CD90.1^+$ TCR-β^+ $CD4^+$), **(c)** B cells (TCR-β^- $CD19^+$), **(d)** neutrophils ($CD11b^+$ $Ly6G^+$), **(e)** T cells ($CD90.1^+$ TCR-β^+), and **(f)** $CD8^+$ T cells ($CD90.1^+$ TCR-β^+ $CD8^+$). In the lamina propria, CTx altered the frequency of **(g)** regulatory T cells (Treg) ($CD90.1^+$ TCR-β^+ $CD4^+$ $Foxp3^+$), **(h)** dendritic cells ($CD11c^+$ MHC-II^+), **(i)** Th17 cells ($CD90.1^+$ TCR-β^+ $CD4^+$ $CD44^+$ RORγT^+), and **(j)** RORγT^+ Treg cells ($CD90.1^+$ TCR-β^+ $CD4^+$ $Foxp3^+$ RORγT^+). All populations were gated on $CD45^+$ live cells. Data are presented as mean ± s.e.m. Statistical significance was determined using unpaired two-tailed *Student's t*-test. * $p \leq 0.05$, ** $p \leq 0.01$, *** $p \leq 0.001$. Data are pooled from two independent experiments.
