# Supplementary material for: Cholera toxin remodels intestinal immunity and suppresses systemic metabolism
Source: bioRxiv. 2026 Oct 6:2026.09.29.755509. Preprint. [Version 1] doi: 10.64898/2026.09.29.755509 (PMC13691948; doi:10.64898/2026.09.29.755509)
Supplement: Supplementary file 7 [file media-7.pdf]

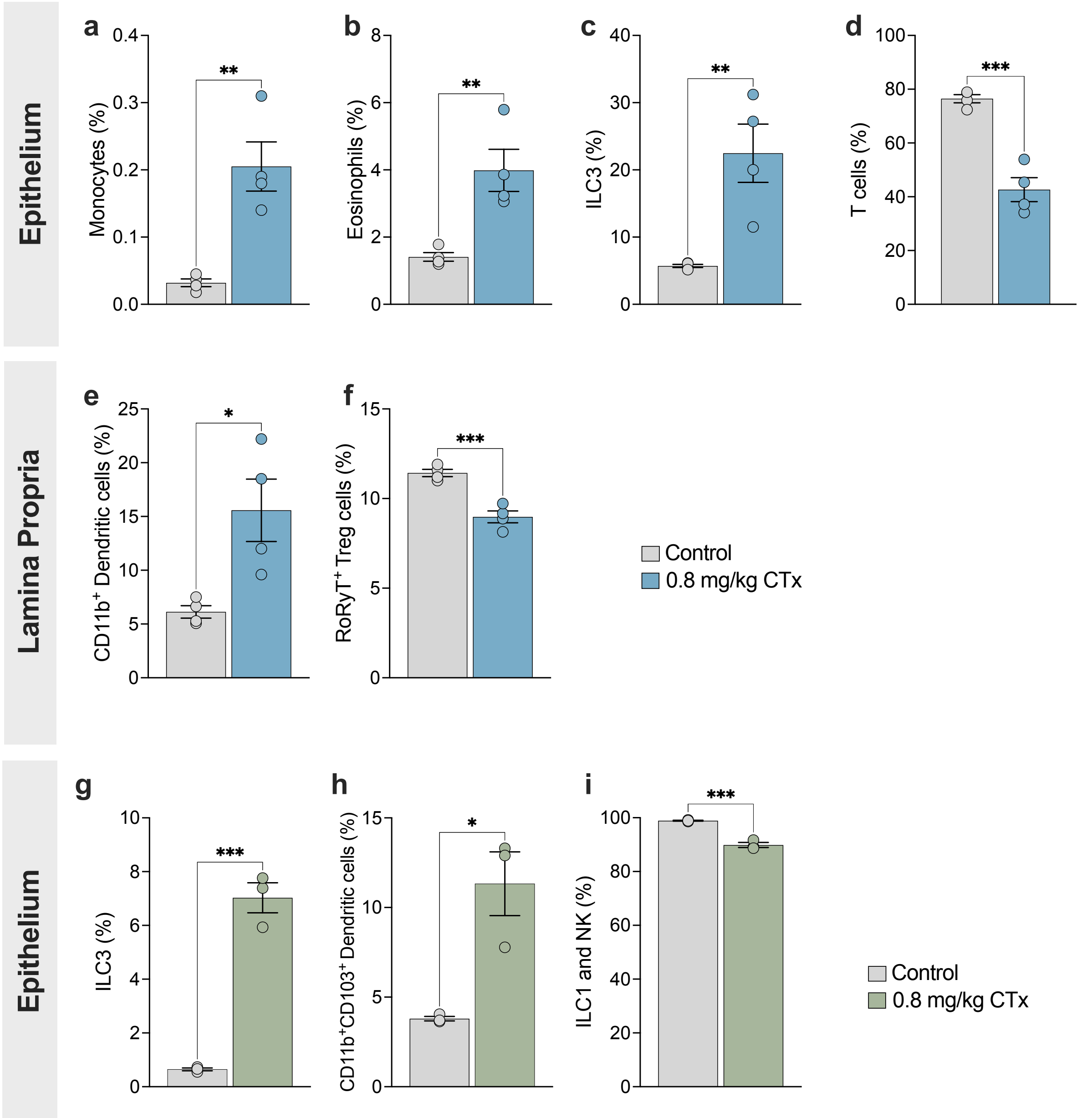

Supplementary Figure 6 | CTx induces modest changes in immune cell frequencies in the small intestine of mice. Small intestines from BALB/c female and male mice were collected 24 h after a single oral exposure to cholera toxin (CTx), enzymatically dissociated, and analyzed by full-spectrum flow cytometry. In females, CTx altered the frequency of **(a)** monocytes (CD11b$^-$ F4/80$^-$ Ly6C$^+$), **(b)** eosinophils (CD11b$^+$ SiglecF$^+$), **(c)** ILC3 (CD90.1$^+$ TCR-β$^-$ TCR-γδ$^-$ RORγT$^+$), and **(d)** T cells (CD90.1$^+$ TCR-β$^+$). In the lamina propria, CTx increased **(e)** CD11b$^+$ dendritic cells (CD11c$^+$ MHC-II$^+$ CD11b$^+$), and decreased **(f)** RORγT$^+$ regulatory T cells (Treg) (CD90.1$^+$ TCR-β$^+$ CD4$^+$ Foxp3$^+$ RORγT$^+$) (n= 4 mice per group). In males, CTx increased the frequency of **(g)** ILC3 (CD90.1$^+$ TCR-β$^-$ TCR-γδ$^-$ RORγT$^+$), **(h)** CD11b$^+$ CD103$^+$ dendritic cells (CD11c$^+$ MHC-II$^+$ CD103$^+$ CD11b$^+$), and **(i)** ILC1 and NK (CD90.1$^+$ TCR-β$^-$ TCR-γδ$^-$ GATA3$^-$ RORγT$^-$) (n= 3 mice per group). All cell populations were gated on CD45$^+$ live cells. Data are presented as mean ± s.e.m. Statistical significance was determined using unpaired two-tailed *Student's t*-test comparing control and CTx-treated groups. * $p \leq 0.05$, ** $p \leq 0.01$, *** $p \leq 0.001$. Data are pooled from two independent experiments.
